# Association of Controlled Physical Activity with Weight Loss and Less Limitations for Hypertensive Patients

**DOI:** 10.3390/sports13040124

**Published:** 2025-04-17

**Authors:** Roxana Cristina Rad Bodan, Adina Octavia Dușe, Eniko Gabriela Papp, Răzvan Marian Melinte, Minodora Andor

**Affiliations:** 1 Doctoral School, “Victor Babeș” University of Medicine and Pharmacy Timișoara, No.2 Eftimie Murgu Square, 300041 Timisoara, Romania; 2 Department of Balneofiziokinetotherapy and Medical Recovery, Faculty of Medicine, “Dimitrie Cantemir” University Târgu-Mureș, No.3-5 Bodoni Sandor, 540545 Târgu-Mureș, Romania; eni_papp@hotmail.com; 3 Multidisciplinary Heart Research Center, “Victor Babeş” University of Medicine and Pharmacy, 2 E. Murgu Square, 300041 Timisoara, Romania; andor.minodora@umft.ro; 4 Department of Physical Medicine, Balneology and Rheumatology, Faculty of Medicine, “Victor Babeș” University of Medicine and Pharmacy Timisoara, No.2 Eftimie Murgu Square, 300041 Timisoara, Romania; duse.adina@umft.ro; 5 Department of Orthopedics and Traumatology, Iuliu Hațieganu” University of Medicine and Pharmacy Cluj-Napoca, No. 8 Victor Babes, 400012 Cluj-Napoca, Romania; razvanmel@xnet.ro; 6 Department of Internal Medicine I, “Victor Babeş” University of Medicine and Pharmacy, Timişoara, No.2 Eftimie Murgu Square, 300041 Timisoara, Romania

**Keywords:** hypertension, BMI, waist circumference, exercises, hydrotherapy

## Abstract

Background: The overweight population is a major public health problem which is typical for the 21st century, considering the peak of the noncommunicable diseases (NCDs). The connection between hypertension—the number-one risk factor of cardiovascular diseases (CVDs)—and the body mass index (BMI), which is growing worldwide, needs to be taken into consideration. Methods: Four homogeneous groups of twenty-five patients each with hypertension degree 1 benefited from different 8-week recovery programs: recommendation for a healthy lifestyle (all groups—A, B, C and D), antihypertensive medication (groups B, C and D), physical therapy program (group C), and hydrotherapy program (group D). Four parameters were pursued: body mass index (BMI), waist circumference (Wcir.), and systolic and diastolic blood pressure (SBP-DBP). Results: Intragroup comparison between initial and final testing registered a statistically significant decrease in all parameters for group C: BMI (*p* = 0.001), Wcir, SBP and DBP (*p* < 0.0001). Additionally, parameters of group D decreased significantly: BMI (*p* = 0.0005), Wcir, SBP and DBP (each *p* < 0.0001). Group A registered a statistical increase in the DPB parameter (*p* = 0.03), and group B had a significant decrease in SBP (*p* = 0.03). Conclusions: Implication in established physical therapy and hydrotherapy had a better outcome in diminishing all four parameters compared to the recommendations for a healthy lifestyle when patients had to improve their lifestyle by themselves, unsupervised.

## 1. Introduction

In the last 10 years, medical science has placed hypertension on the top of the list of cardiovascular risks, with hyperlipidemia and diabetes second and third, which are linked, in most cases, to nutritional habits; the fourth and fifth are obesity and bad diets, and then the last is smoking [1,2,3]. Not more than five years ago, statistics showed that Europe had 30 million patients with CVDs, and the estimated number of new cases for each year passing is 13 million [4].

Almost 30 years ago, cardiovascular diseases were described as an epidemic for developing countries, and today they are presented as a global burden. Because CVDs were named a silent pandemic, the interventions become imminent. Even with all precautions, the number of hypertensive patients is constantly increasing, and it appears to be nearly impossible to stop; by 2015, it reached 1.13 billion globally [5,6,7]. In the 2021 ESC guide, there is no topic focused on hypertension, but we do observe it mentioned in the top risk factors for arterial fibrillation, along with obesity, both marked in red [8]. Three years later, in 2024, the newest guidelines underlined the acute need to intervene from as many directions as possible regarding the hypertensive population [9].

Millions of new cardiovascular patients are registered every year around the world, and approximately 50% of them have hypertension as diagnosis. Today, obesity is more often associated with cardiovascular diseases. BMI has been the first concern among the risk factors in cardiovascular diseases, especially because in some countries, in 2021, 54% of the adult population was overweight or obese. From the perspective of new CVD cases, in 2021, a majority of patients were from urban areas, and also, regarding gender, most of the patients were women, but in rural areas and among men, mortality was more prevalent [10,11,12].

It is alarming that, in the last decade, groundwork showed the connection between modified BMI and high blood pressure even among children and young adults [13,14]. The data show an annual growth rate for obesity between 0.2% and 7.7%, predominant in most countries [15]. The estimated data for the next five years indicate continuously raising values for blood pressure and BMI. Nowadays, the number-one concern is to reduce the mortality rate of NCDs (noncommunicable diseases) in general and specifically in CVDs (cardiovascular diseases) in order to stop the predicted sequence for 2030 [16,17,18] with simple and effective measures which will be easily accepted by populational groups at risk.

Current studies that investigated the effects on physical activities have shown conflicting results of hypertensive patients. A study based on recommendations for physical activity showed no impact from reducing blood pressure values on hypertensive patients [19]. A high-intensity training program registered limited decreases in blood pressure values in hypertensive patients with no scientific relevance, while a study based on a functional training revealed diminished blood pressure values after the program [20,21]. Further research is needed to provide proper intervention for hypertensive patients.

Therefore, this study pursued the impact of four different interventional approaches, i.e., recommendations for a healthy lifestyle, antihypertensive medication, physical therapy program and hydrotherapy, on body mass index, waist circumference and blood pressure values for hypertensive patients.

The main purpose was to evaluate whether changes in the two anthropometric parameters and, implicitly, the applied program will influence the BP values. According to the hypothesis, the 8-week interventional programs are expected to decrease BMI and waist circumference, leading to a decrease in blood pressure values as well. Recognizing the potential importance of training and professional interventions in BMI, Wcir, DBP and SPB can help in the identification of contributing factors and the clinical interpretation of the parameters for hypertensive patients.

## 2. Materials and Methods

### 2.1. Study Design

More than 250 patients diagnosed with degree 1 hypertension were selected from the cardiologist’s records of the county, at the beginning of 2023. Their personal clinical files were analyzed, informed consent was obtained and inclusion criteria were met.

Patients were eligible for the study if they met the following inclusion and exclusion criteria. Criteria that determinate inclusion: (1) age between 30 and 60 years old; (2) hypertension degree 1 with SBP 140–159 mmHg and DBP 90–99 mmHg [22,23], recently cardiology diagnosed and without previous treatments, neither pharmaceutical nor non-pharmaceutical; and (3) written consent signed for both sections ((a) analysis of the medical records; and (b) active implication in the recovery program and assessments during the study). Criteria determining exclusion: (1) pregnant or postpartum period for woman; (2) SBP or DPB values with a higher difference of 15 mmHg between arms; (3) dermatological or other skin pathology that may be contraindicated in physical therapy or hydrotherapy; (4) chronic diseases associated; (5) inflammatory diseases that may influence their physical implication in the recovery program; and (6) medication treatment that may influence the BP values—other than antihypertensive drug treatment recently prescribed, only if required.

The patients meeting the inclusion and exclusion criteria were divided into 4 homogenized groups subsequent to age, and nine homogeneity criteria were fulfilled. Consequently, this prospective interventional study was conducted with 100 patients divided into four homogeneous groups, named A, B, C and D, with 25 individuals each (Table 1).

### 2.2. Intervention

Each of the four groups had a different interventional approach:

→ Group A benefited from recommendations for a healthy lifestyle.
→ Group B, besides counseling for a healthy lifestyle, started administering antihypertensive medication prescribed by the cardiologist.
→ Group C had both interventions described previously, i.e., healthy lifestyle counseling and medication, along with an interventional program based on physical therapy.
→ Group D received both the interventions that were previously described, i.e., healthy lifestyle counseling and medication, along with an interventional program based on hydrotherapy.

#### 2.2.1. Recommendations for a Healthy Lifestyle

The counseling based on the WHO recommendation aims to improve health by explaining the behavioral risk factors for the cardiovascular system that need to be reduced or excluded if possible. All patients from groups A, B, C and D were instructed to fulfill changes in four directions: unhealthy diet, sedentarism, smoking and alcohol consumption.

Regarding diet, the number-one concern was the salt consumption, which was recommended to be diminished as much as possible. Processed food was highly recommended to be substituted with fresh vegetables, fruits, white meat and fish. Adequate water intake, in addition to avoiding juice and especially fizzy drinks, was also recommended.

Smoking needs to be reduced or excluded if possible, taking into consideration the patient’s addiction, type and number of cigarettes per day in order to estimate the nicotine withdrawal timeline for these patients. We strongly recommend avoiding alcohol, especially the refined type; for optimal results, abstinence for several weeks, during the implemented program, is recommended.

Sedentarism is a constant struggle that needs to be combatted. Recommendations sought to initiate more physical activity in their free time or at work, if possible, in order to have moderate activity daily and 2–3 sessions of intense exercise weekly. If none of this is possible, due to lack of time or interest in any physical activity, walks are the most beneficial alternative, combined with gardening or cycling. An observation needs to be made regarding life nowadays: daily chores are no longer considered physical activity [24,25].

#### 2.2.2. Antihypertensive Medication

Based on patients’ anamnestic data, the cardiologist prescribed the most suitable antihypertensives for the patients of groups B, C and D. The latest studies’ outcomes sustain prescription of pharmaceutical therapy for lowering blood pressure, based on the practical algorithm that sustains starting the treatment with angiotensin-converting enzyme inhibitor (ACEi) or angiotensin receptor blocker (ARB) in combination with diuretics or low-dose non-dihydropyridines calcium channel blockers [26].

#### 2.2.3. Physical Therapy Program

Twenty sessions of physical activity coordinated by specialists in kinetic recovery were implemented for group C. There was an 8-week program conducted in group sessions of 5 to 8 patients, with a frequency of 2–3 sessions weekly. Each session was organized following kinesiotherapy criteria: preparation of the body for the effort with static stretching exercise (10–15 poses sustained for 30 s each), active part with progressive overload constituted in a varied circuits type and adequate recovery post-exercises. From the total time of a session, 50–70 min, the main active part may reach up to 45 min in order to enhance cardiovascular endurance. This dominant section was subdivided into two parts: one represented by a circuit with varied exercise and another one compounded by cycling or walking on a treadmill, using an individualized speed and time ratio for each patient [27,28].

#### 2.2.4. Hydrotherapy Program

Hydrothermal therapy, implemented for group D, consists of 20 sessions of 50–70 min each, with a frequency of 2 to 3 meetings weekly for 8 weeks in a row. To prepare the body for exertion, a few techniques from the physiotherapy section were chosen, i.e., electrotherapy, magnetotherapy, four cellular galvanic baths, paraffin and mud applications. The predominate active part reaches up to 45 min, and it is represented by exercise in the pool combined with walking and climbing stairs by using the resistance of water at different submersion levels. The recovery after the effort was also carried out in the pool, under the recommendations of freestyle swimming and flouting. All patients benefited from floating devises during practice in order to keep them safe and secure [29].

### 2.3. Evaluation

Participants who were enrolled were required to visit the laboratory twice during the prospective interventional study: first, for an initial assessment, before implementing the program; and then for a final assessment after 8 weeks of the predetermined program.

All visits were performed during the same daytime hours, in the afternoon, between 14 and 15 to avoid differences that may be induced by diurnal variation. Upon arrival at the laboratory, the anthropometrics measurements of the patients—BMI and waist circumference—were obtained by trained research assistants respecting standardized protocols. For height measurement, a moveable anthropometer was used, and patients were in an orthostatic position, with bare feet on a flat surface, with the weight equally distributed on both feet and the head in the Frankfort Horizontal Plane. A certified standard scale was used with accuracy within 50 g [30].

Monitoring and measuring blood pressure took place in the same conditions for all patients, taking into consideration the impact of the white-coat phenomenon and the training needed for both patients and specialists. Several measurements have been made to obtain the most accurate value: blood pressure measurement is made after 5 min of physical and psychological rest; three measurements in a row are made, and the value obtained from the arithmetic average of the last two is written down [31,32].

BMI formula and classification did not change over time and remain an important parameter for the health in general and for the morbidity risks: BMI = weight (kg) ÷ height^2^ (meters). Interpretation BMI: underweight, <18.5 kg/m^2^; healthy, 18.5–24.9 kg/m^2^’ overweight, 25–29.9 kg/m^2^; obesity, 30–39.9 kg/m^2^; and severe obesity, >40 kg/m^2^ [33,34].

For waist circumference measurements, a flexible steel tape calibrated in centimeters within millimeter graduation will be placed comfortably but still tight around the lateral aspect of each ilium at the mid-axillary line, with the patients standing with their arms crossed on opposite shoulders from the orthostatic position [35].

Waist circumference has gained its place alongside BMI as an indicator that comes to underline potential cardiovascular risks. Studies suggest that BMI analysis completed with waist circumference will bring a superior view regarding obesity in general, but mostly in correlation with cardiovascular diseases. Optimal waist circumference is <94 cm for men and <80 cm for women; measurements that register a waist circumference higher than 102 cm for men and higher than 88 cm for women are corelated with a major cardiovascular risk [36].

### 2.4. Statistical Analysis

Statistical analysis was performed using GraphPad Prism V.9. To evaluate the data normality, the Shapiro–Wilk test was used. To make comparisons between groups’ data with a normal distribution, we used Student’s *t*-test for dependent data, and the Wilcoxon test when data were not normally distributed. In both cases, in order to compare the initial result with the final ones within each group, the outcomes with a *p* ≤ 0.05 were considered statistically significant.

For the comparison between multiple groups, we used the ANOVA test for numeric values with normal distribution and the Kruskal–Wallis test for values not normally distributed. Also, for the post hoc analysis, we applied Tukey’s multiple comparisons test and Dunn’s multiple comparisons test. The results are expressed as arithmetic mean ± standard deviation and as median (IQR). The level of statistical significance was set for *p* ≤ 0.05.

## 3. Results

### 3.1. Initial and Final Intergroup Testing 

In regard to age, the groups had no statistical differences: group A had an age mean of 46.12 ± 7.78, group B had an age mean of 45.96 ± 7.27, group C had an age mean of 46.32 ± 7.50 and group D had an age mean of 46.08 ± 7.83. Additionally, ANOVA analysis disclosed no significant statistical difference among groups, *p* = 0.99.

**Figure 1:**
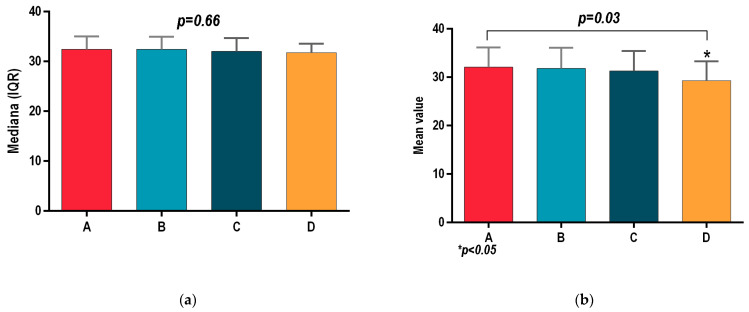
(**a**) Initial assessment of body mass index (BMI) for groups A, B, C and D. (**b**) Final assessment of body mass index (BMI) for groups A, B, C and D. *p* ≤ 0.5 was considered significant.

**Figure 2:**
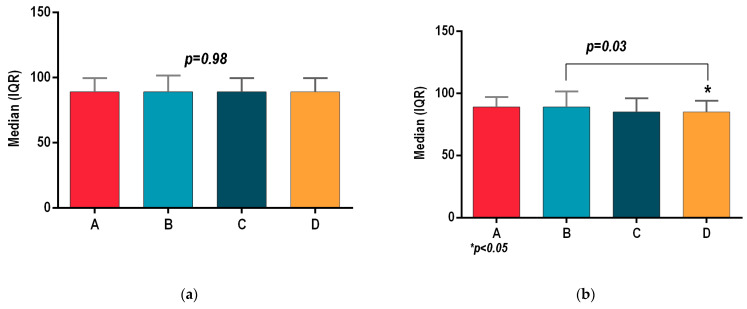
(**a**) Initial assessment of waist circumference for groups A, B, C and D. (**b**) Final assessment of waist circumference for groups A, B, C and D. *p* ≤ 0.5 was considered significant.

**Figure 3:**
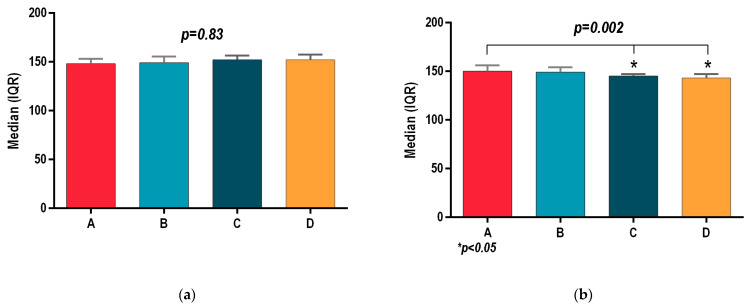
(**a**) Initial assessment of systolic blood pressure (SBP) for groups A, B, C and D. (**b**) Final assessment of systolic blood pressure (SBP) for groups A, B, C and D; *p* ≤ 0.5 was considered significant.

**Figure 4:**
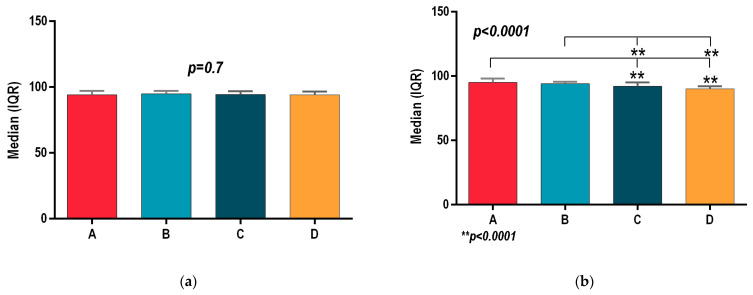
(**a**) Initial assessment of diastolic blood pressure (DBP) for groups A, B, C and D. (**b**) Final assessment of diastolic blood pressure (DBP) for groups A, B, C and D. *p* ≤ 0.5 was considered significant.

All patients of groups A, B, C and D registered similar outcomes at the initial assessment, with no statistically significant differences for body mass index (BMI *p* = 0.66; Figure 1a), waist circumference (Wcir *p* = 0.98, Figure 2a), and systolic and diastolic blood pressure (SBP, *p* = 0.83; DBP, *p* = 0.7; Figure 3a and Figure 4a). Before the interventional programs, the baseline parameters analyzed using the statistical ANOVA test showed homogeneity among all four groups for all parameters, *p* > 0.05.

Statistically significant differences were observed among groups in all parameters, such as body mass index, waist circumference, and systolic and diastolic blood pressure, when analyzed via the ANOVA test following 8 weeks of the implemented programs (*p* < 0.05).

Figure 1a presents the initial assessment for body mass index, which had no statistically significant intergroup differences: A (32.46 (30.44–35.01)), B (32.45 (29.02–34.97)), C (32 (30.35–34.68)) and (D 31.77 (28.9–33.55)); BMI *p* = 0.66.

Figure 1b shows the body mass index of groups A, B, C and D after 8 weeks of the final assessment, where the best outcome was registered by the hydrotherapy program of group D, with (29.33 ± 3.96), which, compared to group A (32.10 ± 4.07), showed a statistically significant decrease, *p* = 0.03. Group B (32.10 ± 4.07) registered the same values as group A. Group C’s mean (31.27 ± 4.17) was lower than that of groups A and B, and it was higher than the mean of group D, but there are no statistically significant differences between any of them.

Figure 2a shows that the initial assessment for waist circumference (Wcir) had no statistically significant intergroup differences: A (89 (84.5–99.5), B (89 (85.5–101.5), C (89 (85–99.5) and D (89 (85–99.5); Wcir *p* = 0.98.

After 8 weeks of final assessment, Figure 2b presents the second anthropometric parameter, waist circumferences (Wcir), of groups, B, C and D. The hydrotherapeutic program of group D, once again, was the most effective: Wcir = 85 (81–94) when compared to group B, which registered Wcir = 89 (85–101.5) with statistically significant difference *p* = 0.03. The Wcir results of group A, 89 (85–97), were similar to the group B, and the results of group C, 85 (81.5–96), were similar to the results of group D, but there were no statistically significant differences between any of them.

Figure 3a shows that the initial assessment for systolic blood pressure had no statistically significant intergroup differences: A (148 (143.5–151.5), B (150 (145–154.5), C (151 (144–155) and D (150 (144–156.5), SBP *p* = 0.83.

According to the 8-week final evaluation, systolic blood pressure revealed that the hydrotherapy program in group D (143 (140–147) and the physical therapy in group C (144 (141–148) both showed a statistically significant decrease when compared to group A (151 (143–157), SBP *p* = 0,002, Figure 3b. Group B’s outcome registered SBP (144 (141–148) values close to those of group A, but there were no statistically significant differences between them or between group B and groups C and D.

Figure 4a shows the initial assessment for diastolic blood pressure had no statistically significant differences intergroup: A (94 (92–96), B (93 (90–95), C (94 (93–96.5) and (D94 (92–96), DBP *p* = 0.7.

Figure 4b reveals the 8-week outcome of diastolic blood pressure (DBP) for both interventional programs, i.e., physical therapy of group C (92 (90–94)) and hydrotherapy of group D (91 (89.5–92)), indicated a statistically significant difference in the final assessment compared to the other two groups: group A (95 (93–97.5))—benefited from recommendation for healthy lifestyle; and group B (94 (93–96.5))—received antihypertensive medication, along with recommendation for healthy lifestyle; both comparisons showed DBP *p* < 0.0001.

All in all, the hydrotherapy program combined with recommendations for a healthy lifestyle and antihypertensive medication for group D achieved the lowest means and medians for all parameters at the final assessments, when comparing with the other three groups: BMI *p* = 0.03, Wcir *p* = 0.03, SBP *p* = 0.002 and DBP *p* < 0.0001 when compared with groups A and B. The physical therapy combined with recommendations for a healthy lifestyle and antihypertensive medication made group C’s values close to group D’s values: SBP *p* = 0.002 and DBP *p* < 0.0001 when compared with groups A and B. At this point, there was no statistically significant difference between hydrotherapy programs and physical therapy programs.

Furthermore, both groups A and B registered higher means and medians in two different parameters. Group A registered higher values for systolic and diastolic blood pressure, showing that the recommendation for a healthy lifestyle may have an impact on body mass index and waist circumference but not on blood pressure values. Group B recommended a healthy lifestyle in combination with antihypertensive medication that had the highest values for body mass index and waist circumference, indicating that medication has a positive impact on blood pressure values but may not bring any improvements regarding anthropometrics parameters. Nevertheless, there was no statistically significant difference between groups A and B.

### 3.2. Comparisons of Initial and Final Assessment in Intragroup A, B, C and D

Intragroup comparison of initial and final assessments revealed that groups A and B had a statistically significant difference for only one parameter each, while groups C and D had statistically significant differences for all of their parameters (Table 2).

Group A registered a statistically significant increase in DBP with *p* = 0.03, and for the other three parameters, BMI, waist circumference and SBP, the group indicated non-statistically significant values. Group B, which benefited from recommendations for a healthy lifestyle and antihypertensive medication, registered decreased blood pressure values, but only the systolic blood pressure (SBP) was statistically significant, with *p* = 0.03; further, a small decrease in waist circumference meant a slight increase in BMI, but it was not statistically significant (Table 2).

Group C, which, besides receiving recommendations for a healthy lifestyle and pharmaceutical intervention, had a specialized physical recovery program, registered a decrease in all four parameters, with all being statistically significant: BMI (*p* = 0.001), Wcir, SBP and DBP (each had *p* < 0.0001). Group D, which participated in a hydrotherapy program, along with receiving recommendations for a healthy lifestyle and pharmaceutical antihypertensive treatment, achieved a statistically significant decrease in all parameters: BMI had *p* = 0.0005, and each Wcir, SBP and DBP had *p* < 0.0001 (Table 2).

The outcome of group D is to be taken into consideration given the fact that it registered better results compared to all other groups—A, B and C. The mean of BMI dropout was 1.28 points after the 8-week hydrotherapy program—group D; and with 0.73 points after the 8-week physical therapy—group C. The difference noticed for the DBP parameter was a decrease of 3 units mmHg for group D and 2 units mmHg for group C. Regarding the means of waist circumference and systolic blood pressure, both recovery programs registered the same dropout after the 8-week interventions: 4 cm for Wcir and 7 units mmHg for SBP (Table 2).

## 4. Discussion

The study aims to demonstrate BMI and waist circumference reduction with a therapeutic intervention consisting of directions on how to obtain a healthy lifestyle, antihypertensive medication, and specialized programs of physical therapy and hydrotherapy, along with self-control of regular blood pressure values, in an 8-week period for a group of hypertensive patients.

The results of this study show that active implication in established physical therapy or hydrotherapy had a better outcome compared with only recommendation for a healthy lifestyle when the patients needed to do all by themselves unsupervised. Regardless of the expectations, online programs of physical activity proved to be ineffective; for instance, a thermoneutral-water home program did not reach the expected results [37,38]. Therefore, recovery programs based on the direct implication of the trained specialists are most likely irreplaceable.

The recommendation for a healthy lifestyle did not bring any benefits to the patients of group A after the 8-week period; the DBP median, which was higher at the end than the beginning of the implementation of the program, indicated progression of hypertension.

B group, besides receiving the healthy lifestyle recommendations, initiated pharmaceutical treatment, and the results are statistically significant for one parameter: the SBP median was lower after the 8-week program. The cases in which lifestyle changes are related to weight loss and controlled BP values are isolated. At this point, in our study, antihypertensive medication brought additional benefits to the patients that most likely will reduce cardiovascular risks.

The association of medication with physical therapy or hydrotherapy programs had a better outcome for groups C and D in comparison with groups A and B. Both programs brought statistically significant changes for all documented parameters. Once more, it is underlined that systematic and specialized supervised programs are more likely to bring better results in comparison to a free-will program based on theoretical recommendations that the patients need to implement by themselves. Periodical assessments and theoretical recommendations may not be sufficient; a direct implementation and specialized supervision, like in the two recovery-group programs described in this study, represent a more effective path for hypertensive patients.

The current research assessed the impact of four different approaches to controlling the body mass index, waist circumference and blood pressure values of hypertensive patients. The relation between obesity, expressed by high BMI, and hypertension has often been pointed out in studies over the last 5 years [39,40,41]. It is alarming that more studies take into consideration the connection mentioned above, regardless of the age population [42,43]. The interventions are yet to be discussed to determine the best outcome and how hypertension may be prevented, along with achieving a normalized BMI [44,45,46,47].

At this point, the results show that the combination of pharmaceutical and non-pharmaceutical direction should be taken into consideration regarding the treatment of hypertension. Antihypertensive treatments may be postponed when there is no healthy lifestyle implementation before the hypertension diagnostic and if the patients are willing to intervene in this matter. In these cases, the cardiologist will make such a recommendation if there are no high cardiovascular risks and if hypertension is not higher than degree 1. Several national epidemiological studies, SEPHAR I-IV, present the preponderance of arterial hypertension as the main cause of cardiovascular mortality. In 2005, the prevalence of arterial hypertension was higher in rural populations and for men (49.47% countryside vs. 41.58% city and 50.17% men vs. 41.11% women). The prevalence in 2011 was the opposite of the previous data, with most hypertensive patients coming from the city (59.59%), and the number of female patients was higher (54.9%) [48,49]. However, our study focused on the impact of interventional programs upon mixed groups in regard to gender and social environment.

Both physical therapy and hydrotherapy registered significant results, but between the two interventional programs, there are no significant differences at this point. We managed to improve cardiovascular health by increasing calorie burn with these programs based on time efficiency, variety and flexibility. In addition, a full-body workout has an afterburn effect, so calories continue to be burnt even after the session.

An unexpected outcome is to be taken into consideration for the hydrotherapy program, which registered better results for weight loss and diastolic blood pressure compared to all other groups, even compared to the program with physical therapy. Exercises in the pool provide a full-body workout and have a bigger impact on the cardiovascular system without putting stress on the body. If the hydrotherapy program can sustain a better outcome in regard to improving cardiovascular strength, in comparison with the physical program, further investigation and a more detailed analysis need to be performed in order to confirm this theory.

Worldwide, in 2019, around 18.6 million deaths were allocated to CVDs [50]. One year later, in 2020, the numbers increased, reaching 19.1 million deaths due to CVDs. The rise continued, and in 2021, there were 20.5 million registered deaths due to cardiovascular diseases. We must underline the fact that the number-one disease responsible for 60% of CVDs and around 50% of coronary heart disorders is hypertension. Increased BMI was registered for 2.4 million people globally [51]. Intervention is imminent because the predictive statistics data show a continuous increase for BMI, until 2030, for most of the world’s population.

The study has several limitations. Firstly, it used a small sample size of patients (*n*. 25 for each group) from a restricted area. The reason for not including social factors in the baseline was that it would limit the number of participants further; maintaining homogeneity within groups would have been challenging with more criteria included. As a result, an analysis that relied on social factors was not taken into consideration at this point. Our study did not prioritize gender analysis, and as the study progressed, we came to realize that it was not relevant because gender homogeneity was only achieved at the intergroup level; intragroup patients were 64% women and 36% men. While analyzing waist circumference separately for men and women would have been more substantial, our findings indicate that interventional programs have decreased values, resulting in diminished cardiovascular risk. Current research has led to this limitation, but extent research should emphasize gender differences. Additionally, physical and hydrotherapy programs implemented should be tailored to the effort capacity of each gender. Secondly, the 8-week period may be short according to some points of view, especially for the implementation of a healthy lifestyle, where patients had full control over the rate of accomplished tasks. Future studies should have extended follow-ups to sustain this study’s outcomes. Lastly, more parameters may also be pursued regarding overweight and hypertension markers. Despite this limitation, our findings underscore the importance of supervised physical activity programs to control BMI, waist circumference and blood pressure values, thus contributing to lowering CVD risks.

## 5. Conclusions

Our study revealed improvements in BMI, waist circumference and blood pressure values after both physical therapy and hydrotherapy programs. Controlled and guided physical therapy and hydrotherapy had better outcomes compared to the recommendations for a healthy lifestyle alone, even when patients needed to do programs by themselves, unsupervised. The recommendations for a healthy lifestyle as a single method of treatment did not bring significant benefits and is not enough, but when associated with antihypertensive medication, improvements in SBP values were noticed. At this point, we give credit to medication for the progress. Hydrotherapy programs were more efficient in lowering three parameters (BMI, SBP and DBP) than the single physical program, but the results were not statistically significant, so an expendability of the research is needed in this direction to determine which therapeutic intervention or combination of interventions is more effective in lowering the parameters and diminishing cardiovascular risks.

## Figures and Tables

**Table 1:** Patient groups’ baseline subsequent to homogenized criteria.

		A	B	C	D
		*n* (%)	*n* (%)	*n* (%)	*n* (%)
Sex					
	F	16 (64)	16 (64)	16 (64)	16 (64)
	M	9 (36)	9 (36)	9 (36)	9 (36)
Menstruating					
	Not applicable	9 (36)	9 (36)	9 (36)	9 (36)
	Yes	4 (16)	4 (16)	4 (16)	4 (16)
	No	12	12	12	12
Menopause early					
	Not applicable	9 (36)	9 (36)	9 (36)	9 (36)
	Yes	10 (40)	10 (40)	10 (40)	10 (40)
	No	6 (24)	6 (24)	6 (24)	6 (24)
Familial history of HBP					
	Yes	20 (80)	20 (80)	20 (80)	20 (80)
	No	5 (20)	5 (20)	5 (20)	5 (20)
Smoking					
	Yes	7 (28)	7 (28)	7 (28)	7 (28)
	No	18 (72)	8 (72)	8 (72)	8 (72)
Overweight					
	Yes	21 (84)	21 (84)	21 (84)	21 (84)
	No	4 (16)	4 (16)	4 (16)	4 (16)
Active lifestyle					
	Yes	6 (24)	6 (24)	6 (24)	6 (24)
	No	19 (76)	19 (76)	19 (76)	19 (76)
Active work					
	Yes	8 (32)	8 (32)	8 (32)	8 (32)
	No	17 (68)	17 (68)	17 (68)	17 (68)
Difference BP value between arms					
	Yes	4 (16)	4 (16)	4 (16)	4 (16)
	No	21 (84)	21 (84)	21 (84)	21 (84)

A, B, C and D: groups of patients. BP: blood pressure.

**Table 2:** Initial and final assessment, comparison of intragroup A, B, C and D.

	Initial	Final	*p* Value
Group A			
BMI	32.0 ± 4.18	32.10 ± 4.07	0.12
Wcir	91.52 ± 8.92	91.6 ± 8.48	0.7989
SBP	148.6 ± 5.30	149.9 ± 10.12	0.45
DPB	94.16 ± 2.52	95.2 ± 2.73	0.03
Group B			
BMI	32 ± 4.18	32.1 ± 4.07	0.12
Wcir	91.88 ± 8.37	91.80 ± 8.49	0.8184
SBP	149.8 ± 6.18	149 ± 5.62	0.03
DPB	94.72 ± 2.42	94.4 ± 2.44	0.33
Group C			
BMI	32 (30.35–34.68)	31.55 (29.64–33.97)	0.001
Wcir	91.96 ± 8.86	88.72 ± 8.46	<0.0001
SBP	151 (144–155)	144 (141–148)	<0.0001
DPB	94 (93–96.5)	92 (90–94)	<0.0001
Group D			
BMI	31.77 (28.9–33.55)	30.49 (27.89–32.16)	0.0005
Wcir	91.56 ± 8.82	86.56 ± 7.61	<0.0001
SBP	150 (144–156.5)	143 (140–147)	<0.0001
DPB	94 (92–96)	91 (89.5–92)	<0.0001

BMI, body mass index; Wcir, waist circumference; SBP, systolic blood pressure; DBP, diastolic blood pressure; *p* ≤ 0.5 was considered significant.

## Data Availability

The data that support the findings of this study are available on reasonable request from the corresponding author R.C.R.B, upon reasonable request. The data are not publicly available due to privacy and ethical restrictions.

## Abbreviations

The following abbreviations are used in this manuscript:

BMI	body mass index
Wcir	waist circumference
SBP	systolic blood pressure
DBP	diastolic blood pressure
NCDs	noncommunicable diseases
CVDs	cardiovascular diseases
